# Correction to: Whole genome sequencing *Mycobacterium tuberculosis* directly from sputum identifies more genetic diversity than sequencing from culture

**DOI:** 10.1186/s12864-019-5841-8

**Published:** 2019-05-29

**Authors:** Camus Nimmo, Liam P. Shaw, Ronan Doyle, Rachel Williams, Kayleen Brien, Carrie Burgess, Judith Breuer, Francois Balloux, Alexander S. Pym

**Affiliations:** 1 Division of Infection and Immunity, University College London, London, WC1E 6BT UK; 2 Africa Health Research Institute, Durban, South Africa; 3 UCL Genetics Institute, University College London, London, WC1E 6BT UK; 4 Nuffield Department of Clinical Medicine, Oxford University, Oxford, OX3 7BN UK; 5 Clinical Research Department, London School of Hygiene and Tropical Medicine, London, WC1E 7HT UK

**Correction to: BMC Genomics (2019) 20:389**

**DOI: 10.1186/s12864-019-5782-2**

Following the publication of this article [1], the authors reported that one of the authors’ names was typeset incorrectly in the authorship list.

In this Correction article the incorrect and correct author name are shown. The original publication of this article has been corrected.

Originally the author name was published as:

- Rona Doyle

The correct author name is:

- Ronan Doyle

The publisher apologizes to the authors and readers for any inconvenience caused by this error.
