# Supplementary material for: Whole genome sequencing Mycobacterium tuberculosis directly from sputum identifies more genetic diversity than sequencing from culture
Source: BMC Genomics. 2019 May 20;20:389. doi: 10.1186/s12864-019-5782-2 (PMC6528373; doi:10.1186/s12864-019-5782-2)
Supplement: Supplementary file 1 — Supplemetary Methods, Figures and Tables. (PDF 490 kb) [file 12864_2019_5782_MOESM1_ESM.pdf]

Whole genome sequencing *Mycobacterium tuberculosis* directly from sputum identifies more genetic diversity than sequencing from culture

Camus Nimmo, Liam P. Shaw, Ronan Doyle, Rachel Williams, Kayleen Brien, Carrie Burgess, Judith Breuer, Francois Balloux, Alexander S. Pym

Supplementary Material

Supplementary Methods	Page 2
Supplementary Figures	Page 4
Supplementary Table	Page 7

Supplementary Methods

Microbiology

The solid agar proportion method was used to perform phenotypic DST for Durban samples. DST was done for isoniazid (minimum inhibitory concentrations of 0.2µg/ml [low-level resistance] and 1.0µg/ml [high-level resistance]), rifampicin (1.0µg/ml), ethambutol (7.5µg/ml), streptomycin (2.0µg/ml), ofloxacin (2.0µg/ml) and kanamycin (6.0µg/ml).

Bioinformatic analysis

Command line parameters used were as follows:

```
trim_galore = Trim Galore v0.4.4
bbmap = BBMap v38.32
picard = Picard Tools v1.13
qualimap = Qualimap v2.21
freebayes = FreeBayes v1.2
vcffilter and vcfintersect from vcflib v1.0
samtools = Samtools v1.9
varscan = VarScan v2.4.0

####Trimming
echo "Trimming"
$trim_galore --length 50 --no_report_file --paired $file1 $file2

####Build BBMap index
$bbmap ref=$reference

###########BBMap align reads
echo "Mapping reads"
$bbmap -Xmx20g in=$file1trim in2=$file2trim out=$sample.aln.sam ref=$reference t=16
minid=0.98 pairedonly=t pairlen=500 slow=t statsfile=$sample'.stats'

########### Sort sam file
echo 'Sorting .sam file to .bam file...'
AlignmentBAM=$sample.sorted.bam
java -Xmx10g -jar $picard SortSam I=$sample.aln.sam O=$AlignmentBAM
SORT_ORDER=coordinate MAX_RECORDS_IN_RAM=1000000

########### Remove duplicates
echo 'Removing duplicates...'
DeduplicatedBAM=$sample.dedup.bam
Metrics=$sample.metrics.txt
java -Xmx10g -jar $picard MarkDuplicates REMOVE_DUPLICATES=true I=$AlignmentBAM
O=$DeduplicatedBAM METRICS_FILE=$Metrics
```

```
############ Add name to read group
mkdir BBMap098_new
DeduplicatedBAM1='BBMap098_new/'$sample'.dedup.1.bam'
java -Xmx10g -jar $picard AddOrReplaceReadGroups LB=BRC PL=illumina PU=Mtb
SM=$sample I=$DeduplicatedBAM O=$DeduplicatedBAM1

############ Sort bam file
java -Xmx10g -jar $picard BuildBamIndex INPUT=$DeduplicatedBAM1

############ Quality checking
mkdir qualimap_098new
$qualimap bamqc -bam $DeduplicatedBAM1 -outdir qualimap_098new/$sample

############# FreeBayes SNP calling, filter mapping quality at 30 and base quality at 30,
filter PPE genes with vcfintersect
echo 'FreeBayes SNP calling...'
mkdir Variants_DOWN_BBMAP098
FreeBayes_permissive_VCF='Variants_DOWN_BBMAP098/'"$sample"'.vcf'
$freebayes -f $reference -p 1 -m 30 -q 30 -C 10 -b
'BAMs_098_DOWN/'$sample'.down.bam'| $vcfintersect -b $PPEgenes -v | $vcffilter -f "SAF
> 0 & SAR > 0 & AO > 3 & RPL > 0 & RPR > 0"> $FreeBayes_permissive_VCF;
$vcfintersect -b $PPEandRNA -v $FreeBayes_permissive_VCF >
$FreeBayes_permissive_VCF'.noRNA'

############# Filter heterozygous alleles
FreeBayes_het_VCF='Variants_DOWN_BBMAP098/'"$sample"'.FreeBayes.het.vcf'
$vcffilter -f "SRF > 0 & SRR > 0 & RO > 3" $FreeBayes_permissive_VCF >
$FreeBayes_het_VCF
$vcfintersect -b $PPEandRNA -v $FreeBayes_het_VCF > $FreeBayes_het_VCF'.noRNA'

############# Calling variants with VarScan for consensus sequence
mkdir Consensus
echo "Making varscan file..."
$samtools mpileup -B -f NC_000962.3.fasta --positions
PE_PPE_RNA_INVERSE_Comas_190212.bed -q 30 -Q 30
'BAMs_098_DOWN/'$sample'.down.bam' \
| java -Xmx8g -jar $varscan mpileup2cns --min-freq-for-hom 0.95 --min-var-freq 0.95 --min-
coverage 20 --p-value 99e-02 --min-avg-qual 30 > 'Consensus /'$sample'.varscanout'

############# Generate consensus of equal length to reference
echo "Making fasta"
perl varscan_to_pseudoseq.pl 4411532 'Consensus/'$sample'.varscanout' >
'Consensus/'$sample'.fasta'
```

Supplementary Figures

Supplementary Figure 1. Midpoint rooted maximum likelihood phylogenetic tree of all samples. Nodes annotated with bootstrap values. WGS obtained from MGIT are appended _M and those directly from sputum _S.

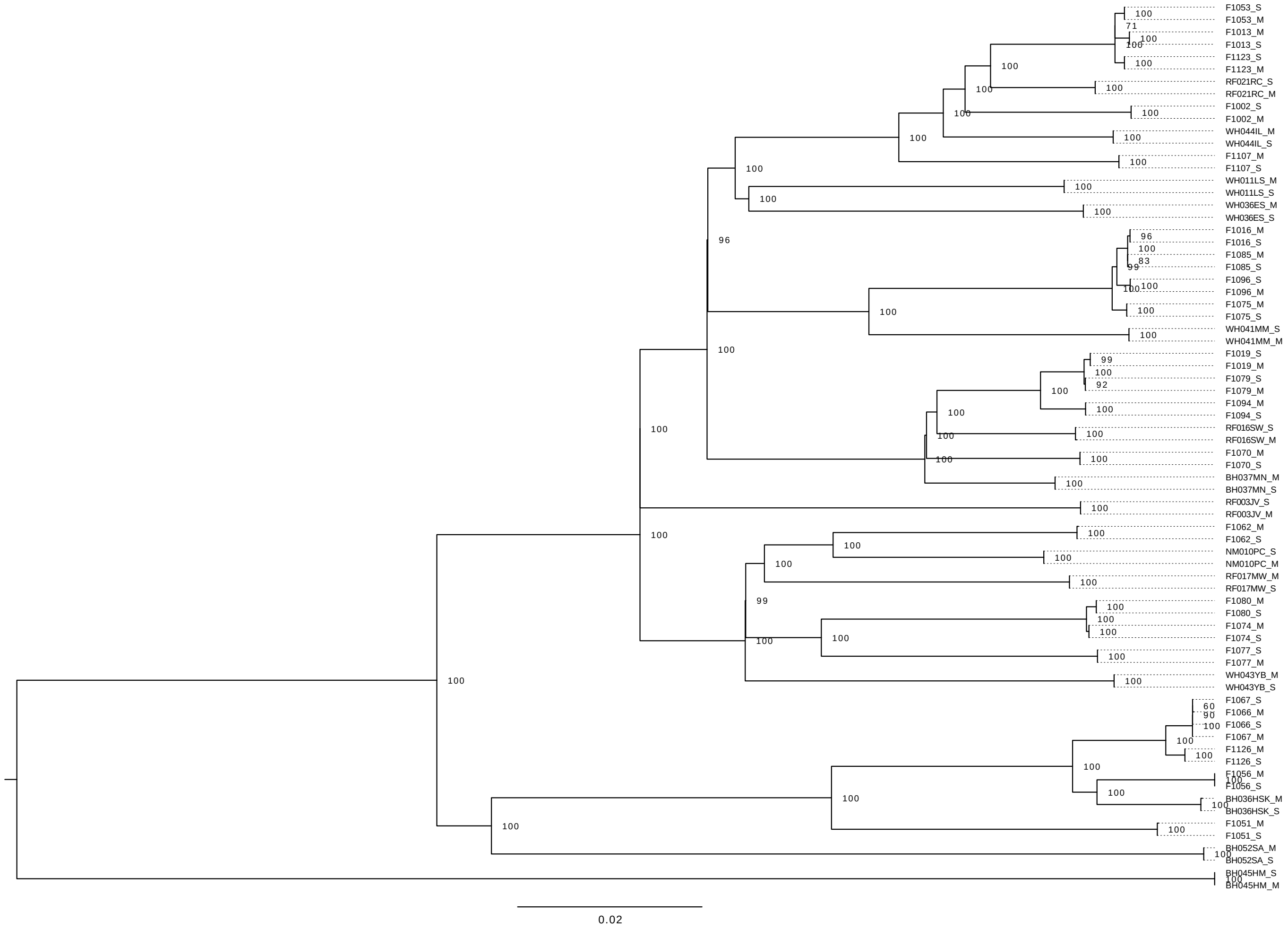

Supplementary Figure 2. Percentage of sampled reads not assigned to *M. tuberculosis* by Kraken (see Methods) that had a blast hit of ≥30 bases to *M. tuberculosis* ribosomal RNA genes (16S or 23S rRNA from H37Rv; see Methods). Bars show median and interquartile range.

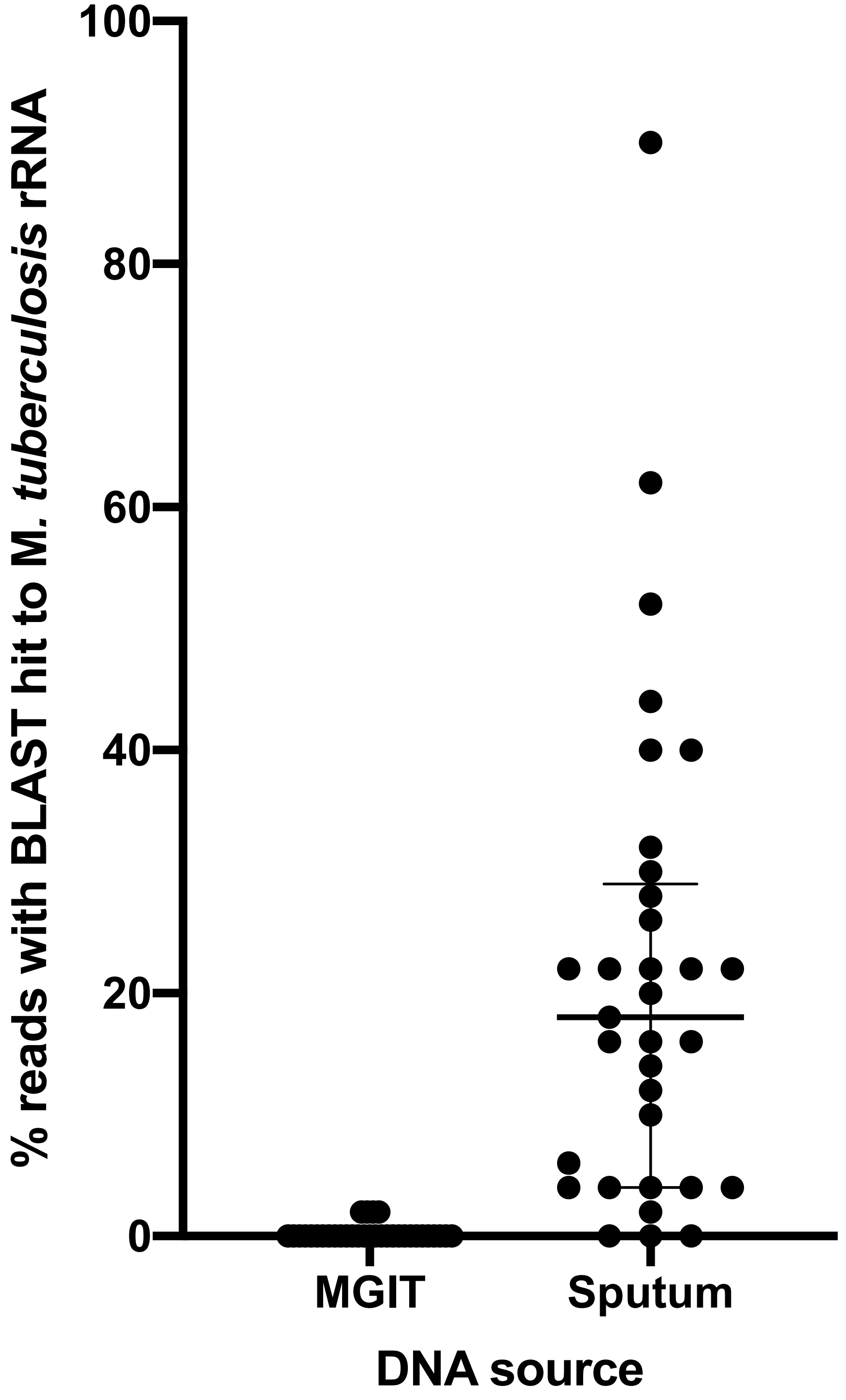

Supplementary Figure 3. Total subsampled reads with a BLAST hit to *M. tuberculosis* ribosomal RNA genes, with colours indicating taxonomic assignment with Kraken (see Methods).

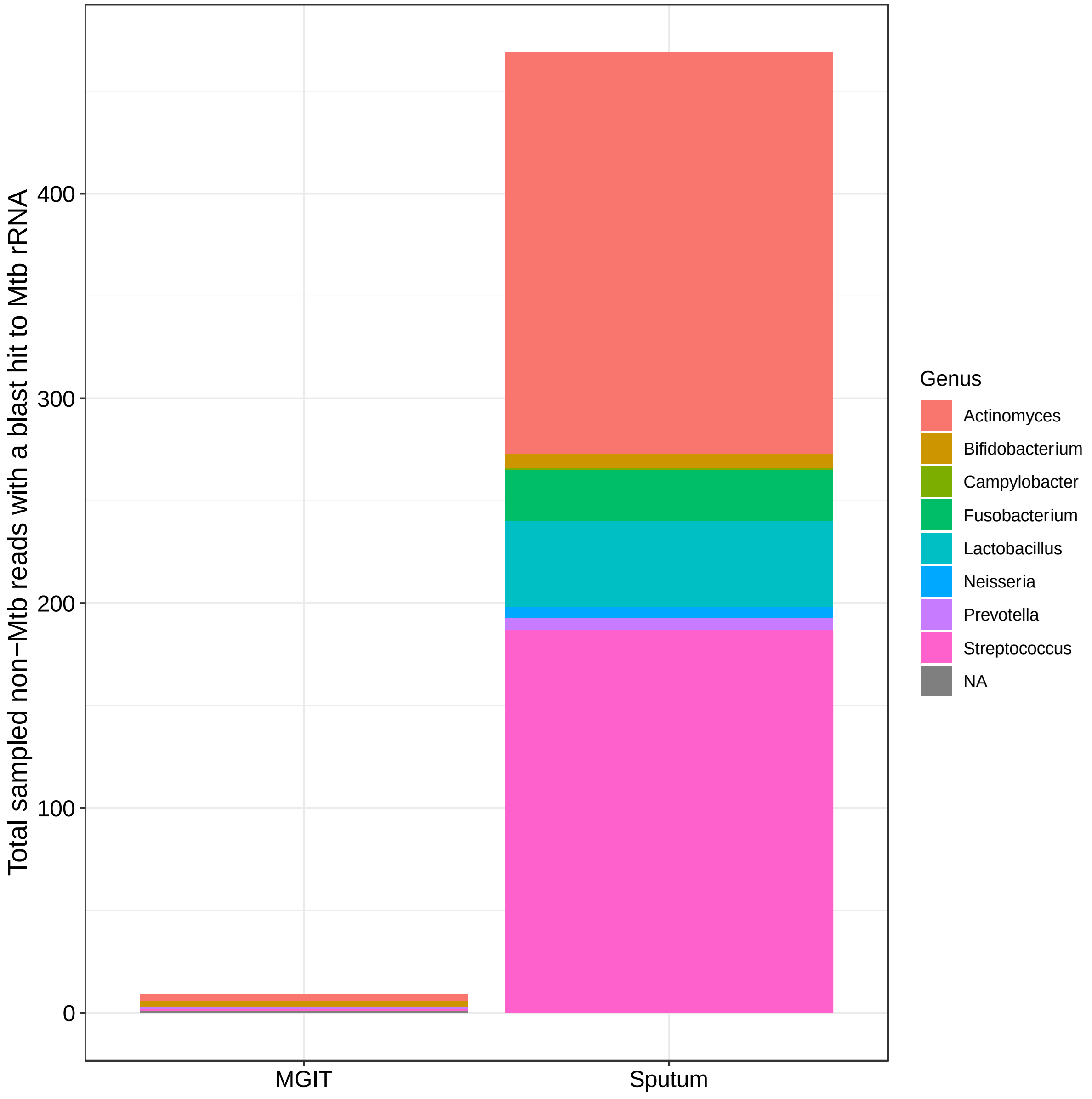

Supplementary Tables

Supplementary Table 1. Original mean coverage depth and number of heterozygous alleles (HAs) after exclusion of hypervariable (e.g. PE/PPE) and ribosomal RNA genes. Patient F1096 had evidence of mixed infection in the sputum sequence and so was excluded from analysis.

Patient ID	Mean coverage depth		% covered at 20x		HA count	
	MGIT	Sputum	MGIT	Sputum	MGIT	Sputum
BH037	71.9	97.3	95.9%	90.3%	1	2
BH052	113.3	244.2	96.6%	90.0%	21	11
F1002	73.9	98.7	96.8%	92.1%	5	6
F1013	96.1	135.0	97.1%	91.6%	2	4
F1016	58.8	255.4	95.6%	96.1%	3	4
F1019	161.5	76.7	97.1%	88.6%	4	6
F1051	241.5	293.4	97.3%	95.6%	7	5
F1053	199.5	216.5	97.8%	95.2%	2	4
F1056	256.1	92.9	96.4%	89.5%	7	12
F1062	123.6	264.2	96.9%	95.9%	8	5
F1066	77.8	563.9	95.8%	96.8%	4	5
F1067	232.4	364.9	95.5%	96.5%	11	7
F1074	184.5	173.7	97.7%	95.1%	3	4
F1075	161.4	101.3	97.2%	90.9%	3	4
F1077	85.4	156.1	95.9%	93.8%	2	4
F1079	118.8	245.2	92.2%	95.7%	4	5
F1080	97.7	94.1	93.5%	90.2%	6	9
F1085	245.6	293.8	97.9%	97.0%	12	10
F1094	87.0	368.5	94.0%	96.7%	5	5
F1096	147.5	60.9	96.7%	85.8%	3	329
F1107	138.0	417.5	96.6%	97.4%	0	1
F1123	124.5	111.1	96.8%	93.1%	2	5
F1126	101.1	314.8	95.8%	96.2%	6	5
NM010	164.9	167.4	98.0%	90.3%	7	7
RF003	139.4	80.4	98.4%	86.7%	5	5
RF016	142.4	314.2	96.7%	97.2%	2	1
RF017	230.1	101.8	97.5%	92.9%	13	13
RF021	147.4	214.1	97.3%	95.8%	11	23
WH011	132.5	172.0	98.4%	92.5%	4	4
WH036	179.3	127.6	97.1%	87.4%	3	20
WH041	188.5	102.2	97.5%	89.2%	0	8
WH043	211.5	286.8	98.4%	97.0%	15	16
WH044	169.0	217.0	98.4%	95.6%	22	45

Supplementary Table 2. Intergenic regions with ≥2 heterozygous alleles (HAs) across all sputum samples, ordered by greatest number of HAs per base.

**Intergenic region**	**HAs per base**		**Total number of HAs**	
	**Sputum**	**MGIT**	**Sputum**	**MGIT**
pe_pgrs18-mprA	0.043	0.022	16	8
pe_pgrs45-rv2616	0.041	0.029	14	10
nrdH-rv3054c	0.040	0.015	19	7
proT-vapC12	0.017	0.003	6	1
alr-rv3424c	0.017	0.003	5	1
pe_pgrs17-rv0979c	0.013	0.000	4	0
rv2355-ppe40	0.009	0.000	7	0
lgt-rv1615	0.007	0.000	5	0
rv0794c-rv0795	0.007	0.005	3	2
rv3428c-ppe59	0.006	0.000	7	0
pe8-rv1041c	0.002	0.001	2	1

Supplementary Table 3. National Center for Biotechnology Information Sequence Read Archive (NCBI SRA) accession numbers for each sample.

Patient ID	NCBI SRA Accession Number	
	MGIT	Sputum
BH037	SRR7725437	SRR7725438
BH052	SRR7725441	SRR7725442
F1002	SRR7725443	SRR7725444
F1013	SRR7725410	SRR7725411
F1016	SRR7725404	SRR7725405
F1019	SRR7725406	SRR7725407
F1051	SRR7725416	SRR7725417
F1053	SRR7725368	SRR7725367
F1056	SRR7725370	SRR7725369
F1062	SRR7725374	SRR7725373
F1066	SRR7725376	SRR7725375
F1067	SRR7725383	SRR7725389
F1074	SRR7725397	SRR7725398
F1075	SRR7725391	SRR7725395
F1077	SRR7725414	SRR7725415
F1079	SRR7725390	SRR7725412
F1080	SRR7725388	SRR7725387
F1085	SRR7725386	SRR7725385
F1094	SRR7725384	SRR7725432
F1096	SRR7725393	SRR7725392
F1107	SRR7725420	SRR7725421
F1123	SRR7725422	SRR7725423
F1126	SRR7725424	SRR7725425
NM010	SRR7725426	SRR7725427
RF003	SRR7725428	SRR7725429
RF016	SRR7725380	SRR7725379
RF017	SRR7725382	SRR7725381
RF021	SRR7725402	SRR7725400
WH011	SRR7725378	SRR7725433
WH036	SRR7725418	SRR7725419
WH041	SRR7725403	SRR7725394
WH043	SRR7725401	SRR7725396
WH044	SRR7725430	SRR7725413
